# Histo- and immunohistochemistry-based estimation of the TCGA and ACRG molecular subtypes for gastric carcinoma and their prognostic significance: A single-institution study

**DOI:** 10.1371/journal.pone.0224812

**Published:** 2019-12-02

**Authors:** Ju-Yoon Yoon, Keiyan Sy, Christine Brezden-Masley, Catherine J. Streutker

**Affiliations:** 1 Department of Laboratory Medicine and Pathobiology, University of Toronto, Toronto, Ontario, Canada; 2 Department of Pathology, St. Michael’s Hospital, Toronto, Ontario, Canada; 3 Department of Hematology/Oncology, St. Michael’s Hospital, Toronto, Ontario, Canada; Universita degli Studi di Verona, ITALY

## Abstract

Gastric cancers comprise molecularly heterogeneous diseases; four molecular subtypes were identified in the cancer genome atlas (TCGA) study, with implications in patient management. In our efforts to devise a clinically feasible means of subtyping, we devised an algorithm based on histology and five stains available in most academic pathology laboratories. This algorithm was used to subtype our cohort of 107 gastric cancer patients from a single institution (St. Michael’s Hospital, Toronto, Canada), which was divided into 3 cases of EBV-positive, 23 of MSI, 27 of GS and 54 of CIN tumours. 87% of the tumours with diffuse histology were classified as GS subtype, which was notable for younger age. Examining for characteristic molecular features, aberrant p53 immunostaining was seen most frequently in the CIN subtype (43% in CIN *vs*. 6% in others), whereas ARID1A loss was rarely seen (6% *vs*. 35% in others). HER2 overexpression was seen exclusively in CIN tumours (17% of CIN tumours). PD-L1 positivity was seen predominantly in the EBV and MSI tumours. As with the TCGA study, no survival differences were seen between the subtypes. A similar strategy was employed to approximate the Asian Cancer Research Group (ACRG) molecular subtyping, with the addition of p53 IHC to the algorithm. We observed rates of ARID1A loss and HER2 overexpression that were comparable to the ACRG study. In summary, our algorithm allowed for clinically feasible means of subtyping gastric carcinoma that recapitulated the key molecular features reported in the large scale studies.

## Introduction

An important cause of cancer-related mortality worldwide, gastric adenocarcinoma (GAc) has long been recognized as a heterogeneous entity, and histo-morphological distinction of the entity into intestinal, diffuse and mixed subtypes is widely practised by surgical pathologists. Comprehensive molecular profiling of 295 primary GAc by the cancer genome atlas (TCGA) proposed four molecular subtypes, namely Epstein-Barr virus (EBV)-positive, microsatellite unstable (MSI), genomically stable (GS) and chromosomal instability (CIN) tumours [1]. Different subtypes were notable for particular molecular pathogenetic features; for example, EBV-positive tumours harboured recurrent *PIK3CA* mutations, along with amplifications involving *JAK2*, *CD274* (encoding PD-L1) and *PDCD1LG2* (encoding PD-L2). The GS subtype was enriched for diffuse histology. While the clustering was robust, reflecting different pathogenetic pathways (ex. EBV infection, genetic and environmental), as well as background genetic factors (i.e. MSI, CIN), the TCGA subtypes did not show significant differences in either overall (OS) or disease-free survival (DFS).

Other molecular subtypes of GAc have been previously described. Lei *et al*. identified three subtypes in their cohort of 70 tumours, termed proliferative, metabolic, and mesenchymal, with survival differences at least in response to surgery with/without 5-fluorouracil [2]. Cristescu *et al*. subcategorized their cohort of 300 GAc (the Asian Cancer Research Group (ACRG) cohort) into four molecular subtypes, and their classification was prognostic across a number of different cohorts examined, including the TCGA cohort [3]. The ACRG classification was based on MSI status, p53, and epithelial-to-mesenchymal transition (EMT) gene expression signature, with the worst survival seen in the microsatellite stable group with EMT signature (MSS-EMT).

Regardless of the prognostic values of these molecular subtypes, incorporating the subtypes into clinical practice is difficult—an unfortunate reality, considering how the different molecular subtypes may call for different treatment strategies. In an effort to devise a clinically feasible strategy, Kim *et al*. had described a ten-stain panel, using EBER, mismatch repair (MMR) proteins (MLH1, PMS2, MSH2, and MSH6), receptor tyrosine kinases (HER2, EGFR, and MET), PTEN, and p53 protein [4]. This panel is extensive, with a number of immunostains (i.e. EGFR, MET, PTEN) that are not routinely utilized in surgical pathology. In order to bring forth a clinically feasible subtyping algorithm, we devised a combination of histology and a five-stain-based algorithm to approximate the TCGA molecular subtypes, utilizing stains available in most academic pathology laboratories. In this study, examining a cohort of 107 GAc cases from a single institution (St. Michael’s Hospital, Toronto, Ontario, Canada), we compared the clinical and molecular features of the approximate molecular subtypes and examined the impact on patient survival.

## Methods

### Tissue microarray and immunohistochemistry

This study was performed in conjunction with our institution’s research ethics board (SMH REB 10–280). We identified cases of gastric adenocarcinoma treated at the St. Michael’s Hospital (Toronto, Ontario, Canada), treated with either gastrectomy or endoscopic mucosal resection (EMR), between the period 2001 to 2011. A tissue microarray (TMA) was constructed as described previously [5], consisting of two 0.6 mm cores per each tumour, with several corresponding normal cores. Histology subtypes were obtained from the pathology reports associated with each case. Diffuse histology was interpreted as per the Lauren classification. Any cases with mixed histology were categorized as “other”.

EBV RNA ISH (EBER) and immunohistochemistry for p53 and MMR proteins were performed according to the standard antibody protocols. Antibodies utilized were: MLH1 (Ventana/Roche M1, pre-dilute antibody), MSH2 (Roche G219-1129, pre-dilute antibody), MSH6 (Cell Marque SP93, concentrated antibody, diluted 1–100), PMS2 (Roche A16-4 pre-dilute antibody), p53 (Roche Pb-53011 pre-dilute antibody), HER2 (Ventana/Roche 4B5), ARID1A (BAF250a, Sigma, St. Louis, MO) and PD-L1 (22C3, Ventana). *In situ* hybridization kits (HER2, Inform Dual ISH Roche/Ventana; EBER, EBER RNA CISH, Roche, pre-diluted antibody) were utilized according to product protocols.

Strong lesional EBER signals were interpreted as positivity for EBV. Mismatch repair (MMR) status was determined using immunostaining for MLH1, PMS2, MSH2 and MSH6. In all cases, the pattern of loss was either the concomitant loss of MLH and PMS2, or concomitant loss of MSH2 and MSH6.

HER2 IHC and ISH were performed as per the clinical guideline on whole sections (where available) or biopsy specimens [6], with dual *in-situ* hybridization (DISH) for *HER2* amplification being used in cases of equivocal/2+ HER2 IHC (Ventana HER2 dual ISH), interpreted by gastrointestinal pathologists with extensive experience in gastric HER2 interpretation. p53 IHC staining was interpreted as being aberrant if either: a) diffusely strong, nuclear staining, or b) complete loss of nuclear staining in all lesional nuclei was identified in tumour cells. ARID1A (BAF250a) was defined as being “lost” if no lesional cells showed any degree of expression; any positivity was interpreted as being “retained”. PD-L1 expression was scored, with any degree of positive staining in greater than 1% of cells scored as “positive”.

### Statistics

Survival analysis was performed using the Kaplan-Meier method. Comparisons of continuous variables between multiple groups were performed using variants of ANOVA. Comparisons of categorical variables between multiple groups were performed using Chi-square test. All statistical tests were performed using JMP (SAS version 13/14).

## Results

### Approximation of the TCGA molecular subtypes

We employed a subtyping algorithm based on the TCGA algorithm, a series of dichotomizing steps. We first identified the EBV-CIMP cases, identified by EBER positivity. The MSI subtypes were next identified through abnormal immunohistochemistry (IHC) for mismatch repair (MMR) pathway proteins, MLH1, PMS2, MSH2 and MSH6, which strongly correlate with MSI-high status [7, 8].

Among the remaining MMR intact, EBER-negative cases, the remainder was subdivided into CIN and GS subtypes. While Kim *et al*. employed a series of five additional markers for this distinction (HER2, EGFR, MET, PTEN, and p53) [4], we chose to distinguish the CIN and GS subtypes based simply on the histology (diffuse *vs*. intestinal/mixed) based on the following rationale: 1) GS tumours were enriched for the diffuse histology in the TCGA study (73% in the TCGA database), with frequent defects in cell-cell adhesion; 2) HER2 and diffuse histology were mutually exclusive in our cohort; and 3) addition of other markers would impose a significant challenge in clinical implementation of a subtyping algorithm.

**Fig 1:**
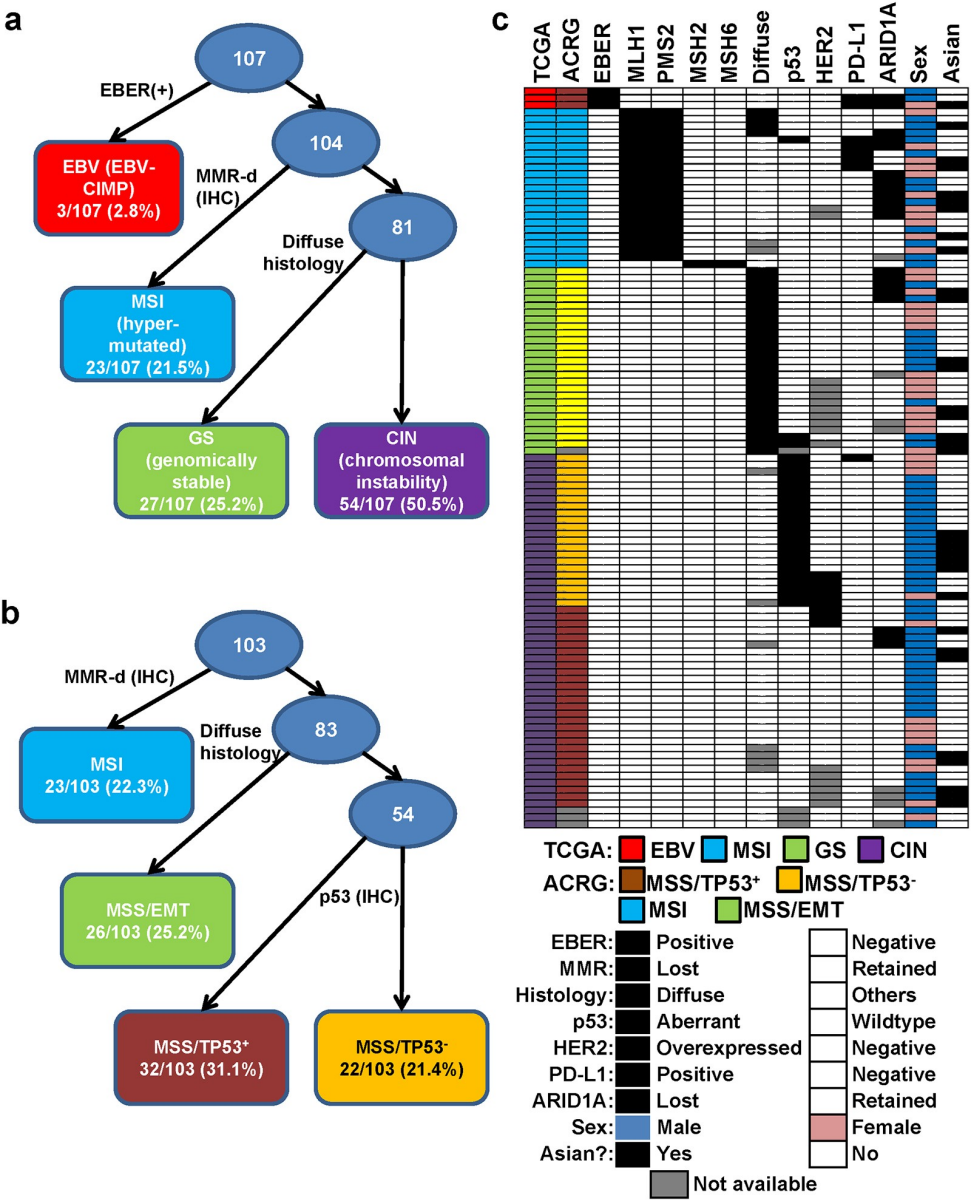
Distribution of gastric adenocarcinoma cases by the approximated molecular subtyping algorithm. A) Subtyping algorithm for approximating the TCGA subtypes. B) Subtyping algorithm for approximating the ACRG subtypes. C) Breakdown of the St. Michael’s Hospital cohort by approximated TCGA and ACRG subtypes, with the IHC/EBER results and clinico-pathological features.

**Fig 2:**
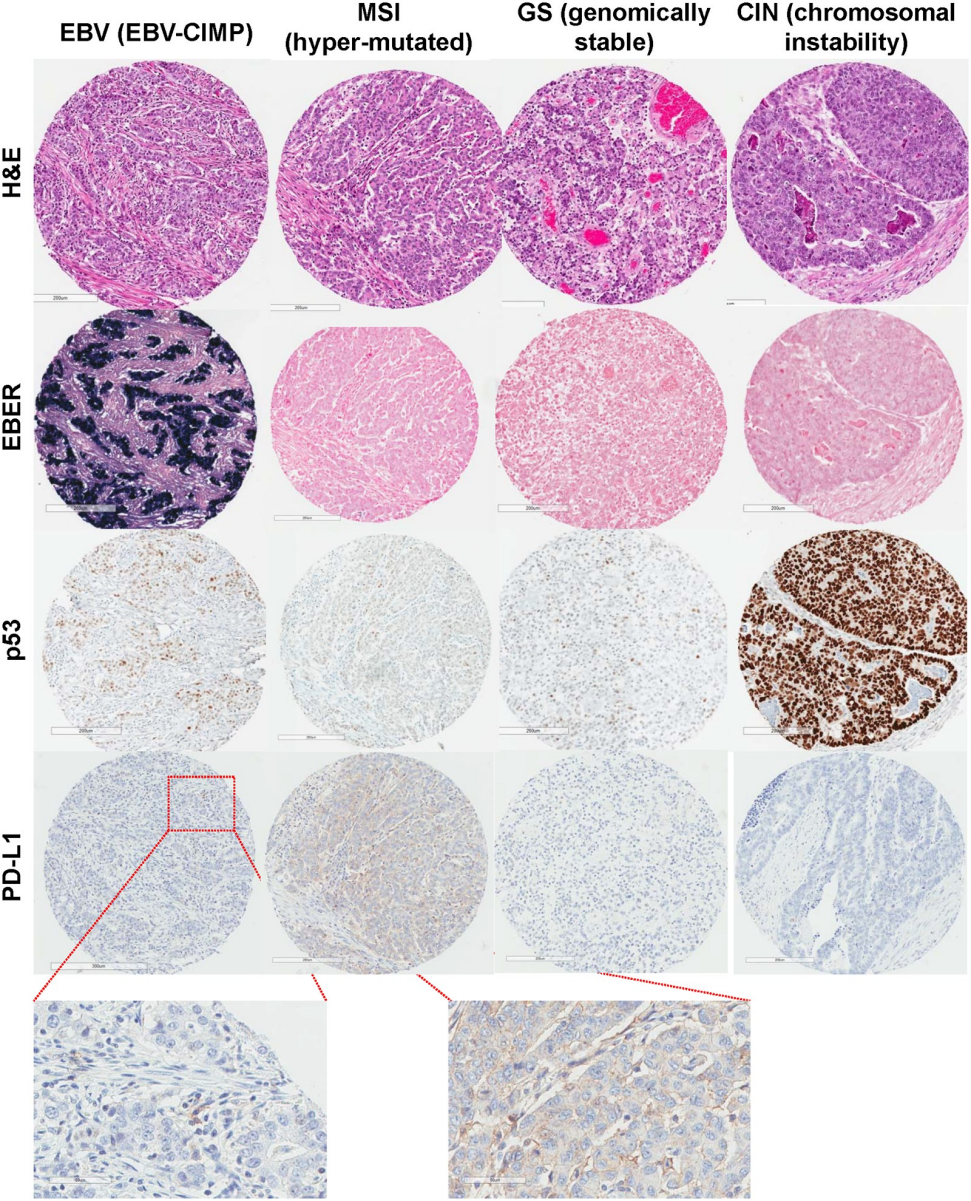
Representative cases of each approximated TCGA molecular subtype.

Using our simplified algorithm, our cohort of 107 GAc cases was divided into three cases of EBV-positive (2.8%), 23 cases of MSI (21.5%), 27 cases of GS (25.2%) and 54 of CIN tumours (50.5%) (Fig 1). 87% of the tumours with diffuse histology were classified as GS subtype; as per the algorithm, all of the GS tumours were of the diffuse histology (compared to 73% in the TCGA study). Representative cases are shown in Fig 2.

The GS tumours were notable for younger age at diagnosis (median 64 *vs*. 70.5 years for others) (Table 1). The median age of diagnosis for MSI tumours was 71 years in our cohort (*vs*. 72 years in the TCGA study). MSI appeared to be slightly enriched in the female patients in our cohort (11 of the 41 tumours from female patients *vs*. 12/66 male patients), with 47.8% of the MSI cases being female patients (*vs*. 56% in the TCGA study). 2/3 EBV-positive tumours were from male patients (*vs*. 81% in the TCGA study), with the one female patient being of East Asian descent. Toronto is home to many residents of East Asian descent, and 30.8% of our cohort was comprised of Asian patients, with no significant differences between the approximated subtypes. Most of the GS patients presented with advanced disease, with the highest number of tumour stage T4 disease at diagnosis (15/27 cases).

**Table 1:** Clinical features of the approximated subtypes in the St. Michael’s cohort.

	EBV	MSI	GS	CIN	p-value (Chi^2^)
**n**	3	23	27	54	
**Median age at diagnosis (range)**	69 (52–79)	71 (32–84)	64 (31–86)	70.5 (19–84)	*p* = 0.3567
**F:M ratio**	1:2	11:12	16:11	13:41	*p* = 0.0136
**Diffuse histology (%)**	0/3	4/23 (17.4%)	27/27 (100%)	0/54	*p* < 0.0001
**Asian (%)**	1/3 (33.3%)	8/23 (34.8%)	9/27 (33.3%)	15/54 (27.8%)	*p* = 0.9206
**T (tumour) Stage**	T1 = 0 T2 = 1/3 T3 = 1/3 T4 = 1/3	T1 = 8/23 T2 = 4/23 T3 = 7/23 T4 = 4/23	T1 = 3/27 T2 = 2/27 T3 = 7/27 T4 = 15/27	T1 = 13/54 T2 = 6/54 T3 = 22/54 T4 = 13/54	*p* = 0.1028
**Node positivity (%)**	1/3 (33.3%)	9/23 (39.1%)	17/27 (63.0%)	34/54 (63%)	*p* = 0.1845
**Advanced disease (%)**	3/3	15/23 (65.2%)	24/27 (88.9%)	41/54 (75.9%)	*p* = 0.1247
**Deaths (%)**	2/3 (66.7%)	4/23 (17.4%)	9/27 (33.3%)	11/54 (20.4%)	*p* = 0.2014

### Molecular features of the approximated molecular subtypes

In the TCGA study, for the non-hypermutated (i.e. *POLE*-wildtype and microsatellite stable) tumours, 25 significantly mutated genes were identified, including *TP53*, *ARID1A*, and *ERBB2* (HER2) [9]. Among them, mutations that showed notable inclinations for specific subtypes were *TP53* (most frequent in the CIN subtype), *CDH1* (more frequent in the GS subtype), *PIK3CA* (EBV-positive), *RHOA* (GS), *ARID1A* (EBV-positive and GS) and *APC* (CIN). 30 focal amplifications were found, with 9p amplifications (containing *JAK2*, *CD274* (which encodes PD-L1) and *PDCD1LG2* (PD-L2)) being enriched in the EBV subgroup (15%).

To compare our approximated molecular subtypes with the TCGA molecular subtypes, we performed immunohistochemistry for p53, HER2, ARID1A and PD-L1. Aberrant p53 (null or aberrantly high) immunostaining was seen most frequently in the CIN subtype (43.1% in CIN *vs*. 6% in others) (Fig 1C and Table 2). As in the TCGA study, aberrant p53 pattern was rarely seen in our GS tumours, being aberrant in only 2/26 of GS tumours (7.7%). The TCGA CIN subtype was also notable for frequent EGFR amplification, including *Erbb2* (HER2) (24% in CIN tumours in the TCGA cohort), and, accordingly, HER2 overexpression was seen exclusively in our CIN tumours (8/48, 16.7%).

**Table 2:** Molecular features of the approximated subtypes.

	EBV	MSI	GS	CIN	Chi^2^
**p53 aberrant (%)**	**0/3**	**1/23 (4.3%)**	**2/26 (7.7%)**	**22/51 (43.1%)** **N/A = 3**	22.098 (*p* < 0.0001)
**ARID1A loss (%)**	**2/3 (66.7%)**	**10/22 (45.5%)** **N/A = 1**	**5/24 (20.8%)** **N/A = 3**	**3/50 (6%)** **N/A = 4**	18.236 (*p* = 0.0004)
**HER2-positive (%)**	**0 (0%)**	**0/21 (0%)** **N/A = 2**	**0/18 (0%)** **N/A = 8**	**8/48 (16.7%)** **N/A = 6**	10.739 (*p* = 0.0132)
**PD-L1 score**	**2/3 (66.7%)**	**5/23 (21.7%)**	**0/25 (0%)**	**1/51 (2.0%)** **N/A = 3**	18.336 (*p* = 0.0004)

**N/A** = not available

This cohort of tumours had previously been examined to report ARID1A loss in about 20% of gastric carcinoma, with the loss being prognostically significant [5]. Our previous study and others had also shown that ARID1A protein loss is more commonly seen amongst MSI cases [5, 10], and, in this study, 10/22 of MSI subtype tumours showed ARID1A loss by IHC. Among the non-MSI-tumours (i.e. the approximated EBV-positive, GS and CIN subtypes), ARID1A protein loss was more frequent in the approximated GS subtype (5/24 of GS cases, 20.8%), in line with the TCGA study results. ARID1A loss was rarely seen in the approximated CIN subtype (3/50, 6%).

We next examined PD-L1. In the TMA, PD-L1 expression was seen only focally in 8 tumours in total. The majority of these cases were EBV-positive (2/3 cases) and MSI (5/23 cases) tumours (Fig 1C). One CIN tumour showed focal PD-L1 positivity.

### Approximation of the ACRG molecular subtypes

The ACRG had classified 300 GAc cases into MSI tumours (22.7%), MSS/EMT tumours (15.3%), MSS/TP53^+^ (26.3%) and MSS/TP53^-^ tumours (35.7%) [3]. As with the TCGA study, the ACRG subtyping was algorithmic in nature, which started with the identification of MSI tumours, followed by further subtyping of the MSS tumours. The worst survival was seen with the MSS/EMT tumours, which were defined based on gene expression profile signature with EMT (epithelial-to-mesenchymal transition) signature. As expected, over 80% of the MSS/EMT tumours were of the diffuse histotypes and presented at younger age, much like the GS molecular subtype in the TCGA study.

The ACRG subtyping was approximated in our cohort, starting with MMR IHC to identify the MSI subtype (Fig 1B). Of the MSS tumours, MSS/EMT subtype was designated based on diffuse histology, and p53 IHC was used to further stratify the remainder (MSS/TP53^+^ *vs*. MSS/TP53^-^). Using this approximated ACRG subtyping algorithm, with 103 cases suitable for subtyping, of which 23 cases (22.3%) were subtyped as MSI, 26 (25.2%) as MSS/EMT, 32 (31.1%) as MSS/TP53^+^ (wildtype p53 IHC) and 22 (21.4%) as MSS/TP53^-^ (aberrant p53 IHC) (Fig 1C). Using this algorithm, all three EBV-positive cases were subtyped as MSS/TP53^+^ tumours. *HER2* gene amplification was restricted to MSS cases in the ACRG study (17.4% in MSS/TP53^-^ and 3% in MSS/TP53^+^) [3], and, in our cohort (where HER2 data was available), 5/22 (22.7%) of the MSS/TP53^-^ and 3/32 (9.4%) of the MSS/TP53^+^ cases overexpressed HER2. *ARID1A* mutations were most commonly seen in the MSI tumours in the ACRG study (44.2%) [3], and ARID1A protein loss was also frequent in the approximated ACRG MSI subtype (10/22, 45.5%). ARID1A loss was seen in other groups although to a lesser extent, specifically 5/26 in MSS/EMT and 5/29 in MSS/TP53^+^ cases. None of the MSS/TP53^-^ cases had lost ARID1A. For PD-L1, the majority of the positive cases were MSI (5/23, 21.7%), but positivity was also seen in MSS/TP53^-^ (1/22) and MSS/TP53^+^ (2/32).

### Patient survival and approximated molecular subtypes

Despite the previously established prognostic significance of the diffuse histology and EBV infection [9, 11–13], the lack of survival differences in TCGA study is rather puzzling. Limited follow-up was proposed as a possible explanation for this lack of difference. In addition, the TCGA cohort was markedly heterogeneous with respect to the patient ethnicity and geographical distribution. These differences are expected to be augmented by the heterogeneity in the clinical care received; similar issues are identified in other large, multi-institution studies.

**Fig 3:**
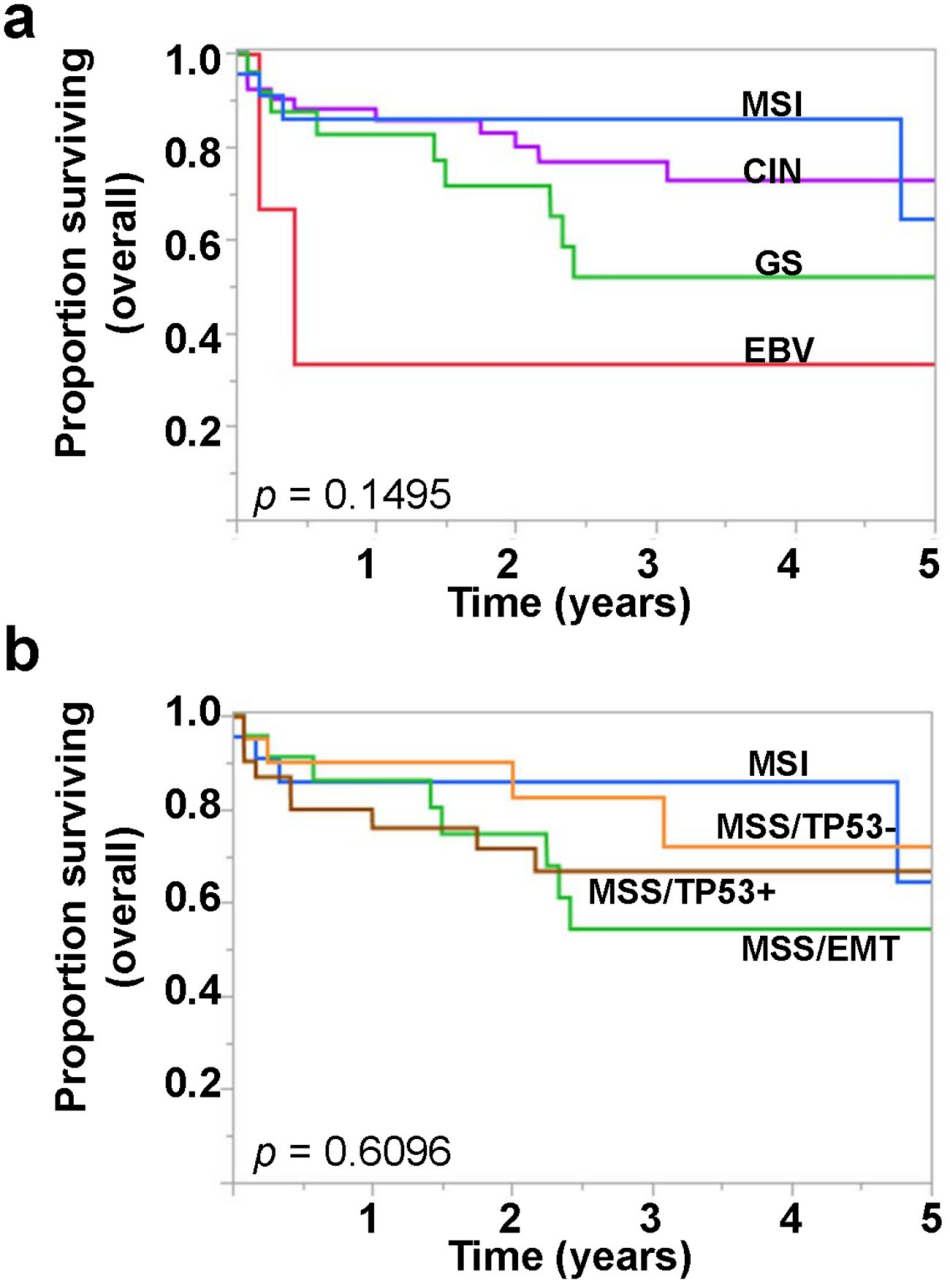
Overall survival of the SMH gastric adenocarcinoma cohort by approximated molecular subtypes. A, B) Overall survival in the SMH cohort grouped by approximated A) TCGA subtypes and B) ACRG subtypes.

We thus examined the prognostic significance of the estimated TCGA subtypes in our cohort. At St. Michael’s hospital (SMH), the gastric adenocarcinoma cohort encompasses some early stage cancers, treated by endoscopic mucosal resection and/or partial gastrectomy. The cohort comprised 33/107 (30.8%) patients of Asian descent, reflecting the rich immigration history in Toronto. Out of 107 patients in our cohort, with a mean follow-up period of 34 months (median 24 months), 26 deaths had occurred during the followup period. In contrast to previously reported association between EBV and better prognosis, 2/3 of EBV-positive patients died early in the disease course (Fig 3a). Among the EBV-negative cases, the worst OS was seen among the approximated GS cases, with 9 deaths (9/27 (33.3%) *vs*. 15/77 (19.5%) for other subtypes). Comparing the GS cases against others, there was a clear separation in the survival curves, but this difference was not significant by log-rank analysis (log-rank *p* = 0.1495). Comparing the approximated GS cases against others, the survival difference remained statistically non-significant (*p* = 0.1696).

We next examined approximated ACRG subtyping with the SMH cohort. As seen with the ACRG study, the approximate MSS/EMT subtype was associated with worse overall survival, with the most number of deaths (8/26 = 30.8%) (Fig 3b). However, the survival differences were small and were not statistically significant (log-rank *p* = 0.6096), likely related to our smaller cohort size.

## Discussion

With recent advances in targeted therapy, surgery with adjuvant chemotherapy is no longer the standard care in many regions, and identification of the proper targeted therapy upfront is an important component in GAc diagnosis. Focusing on routinely available IHC (MMR) and ISH (EBER), we categorized our cohort of gastric cancer patients into “approximated molecular subtypes”. To our knowledge, our study is the first of this kind to approximate the TCGA/ACRG subtypes in a well-mixed (30.8% Asian) cohort (compared to other single institutional studies from Asia or elsewhere ([14, 15]) that received care from a single institution (compared to the multi-institutional TCGA study). To our surprise, no significant survival differences were not observed in our cohort with the approximated TCGA subtyping algorithm, suggesting that the lack of survival differences in the TCGA study may be biological.

Though MSI-associated colonic adenocarcinomas classically have an associated inflammatory infiltrate, this is seen less often in upper gastrointestinal tract adenocarcinomas. Considering the lack of a robust histological marker for MSI phenotype, our data argues for routine MMR testing, as performed in a number of other cancers. MMR status alone was prognostic in the ACRG study [3], and similar findings had been reported in a meta-analysis [16]. Beside its prognostic significance, MSI/MMR deficiency is also becoming an established biomarker in the setting of immune checkpoint inhibition, being now routinely tested in colorectal and endometrial carcinoma [17], and with early results suggesting that MSI gastrointestinal tumours from a wide variety of sites respond to checkpoint blockade [17]. Gonzalez *et al*. had reported a subtyping algorithm based on two IHCs (p53 and MLH1), with EBER [18]. While MLH1 is most commonly lost in MSI-related gastric cancers, one study saw loss of MLH1/PMS2 in only 88% of MSI-H cases [19], and thus a single marker is expected to miss at least 10% of the MMR-deficient cases. Another more recent subtyping study employed EBER, in combination with IHC for p53 and MMR (4 markers) [20]. This method would allow for somewhat of a hybrid subtyping algorithm that combines some aspects of TCGA and ACRG.

As a method for stratifying treatment strategy, should PD-L1 IHC replace EBER in our algorithm? Recently, a modified method for PD-L1 scoring, combined positive score (CPS), which combines PD-L1 expression in both the tumour and immune cells, was described as being a superior method to the conventional tumour proportion score method [21]. While the study reports high inter-observer concordance rate (87.6%), PD-L1 testing remains unavailable in many pathology laboratories. Significant questions still remain about the best antibody to use as well as the appropriate scoring system and where to look within the tumour (advancing front *vs*. superficial) [22]. In our study, PD-L1 positivity was limited to a small number of cases, although positivity was predominantly in the EBV and MSI groups as expected. This may have been due to the age of the blocks, some of which were more than 10 years old. PD-L1 staining has been reported to fade as blocks age [23]. As the study was also done on a TMA, the advancing front of the tumour, often the site of highest PD-L1 staining in gastrointestinal tract cancers is less likely to have been sampled for inclusion in the TMA. The practice of coring tumours or interpreting biopsy specimens are thus expected to introduce a bias and examining whole sections would be ideal in the clinical setting. While interpreting EBER results is not without its own challenges, EBER interpretation is expected to be more reproducible (*vs*. PD-L1), with higher concordance rates for biopsy-resection and primary-metastasis comparisons [24]. In addition, other potential therapeutic targets, such as *PIK3CA* and *JAK2*, may be explored in EBV-positive tumours.

**Fig 4:**
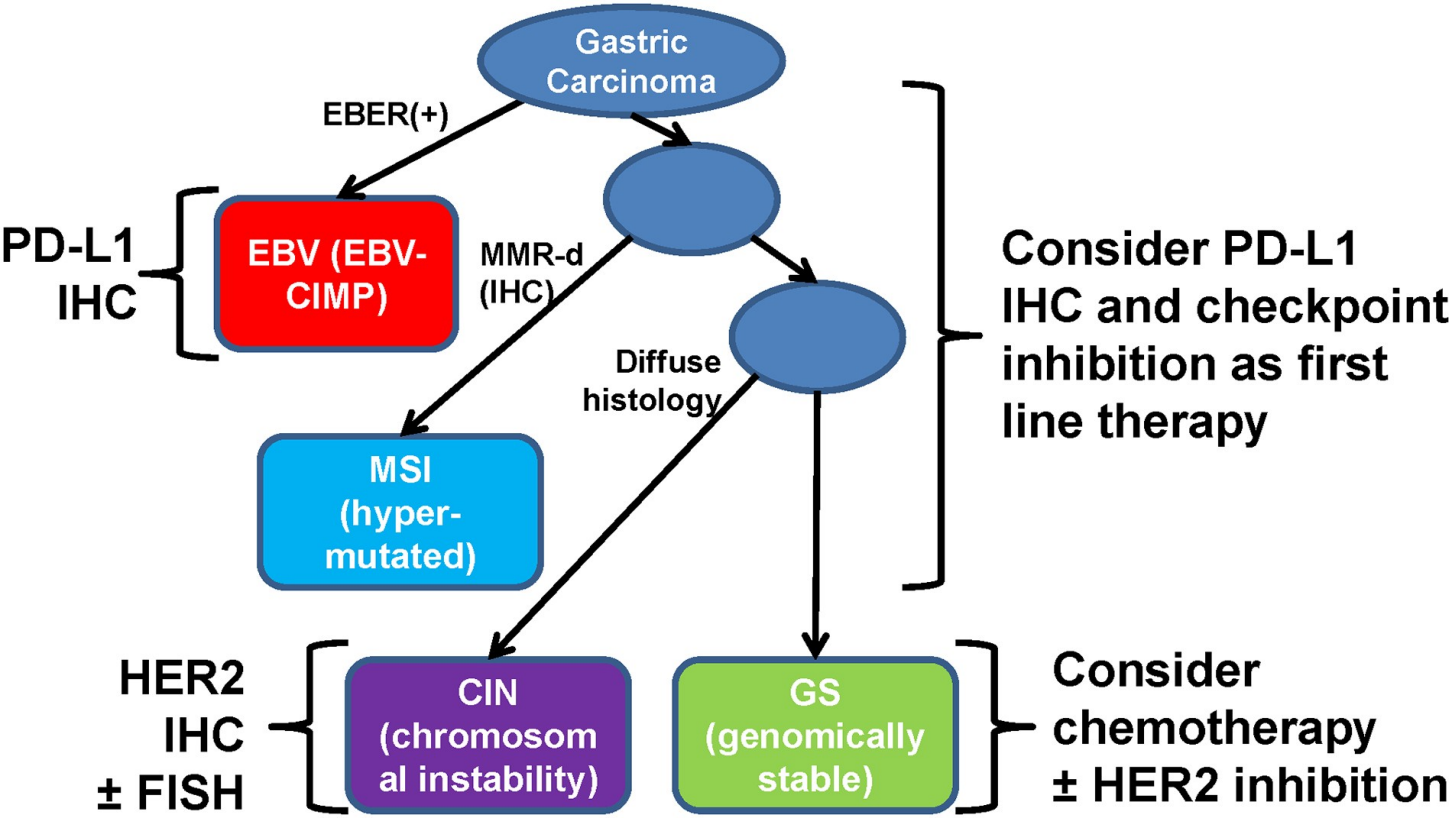
Clinical/laboratory approach to gastric adenocarcinoma and its impact on therapy. Testing and/or targeting PD-L1 should be considered in the EBV and MSI subtype tumours. The non-EBV/MSI tumours should be considered for judicious HER2 testing, based on the histology.

A major weakness of this study is our relatively small sample size, which may be the main factor behind the lack of significant survival difference in approximate ACRG subtypes. We are particularly limited in the number EBER-positive cases, thus limiting our analysis with a prognostically important subgroup. Validating the molecular correlations (including sequencing of genes with expected mutations) was outside the scope of this study, and we are currently lacking a second cohort to validate our findings. Despite these weaknesses, the strength in the approach lies in the clinical feasibility. The current College of American Pathologists (CAP) guideline does not require routine testing of EBV or MMR/MSI. While our algorithm is crude, the five-stain method is a clinically feasible means of recognizing the inherent heterogeneity in GAc, and our subtyping recapitulated some key clinico-molecular features reported in the TCGA study. Beyond simple recapitulation, our algorithm allows for logical approach in strategizing patient treatment and judicious testing for additional biomarkers, as suggested in Fig 4. Gastric cancer remains a highly fatal disease with 10,800 deaths estimated in the US (2018 SEER) [25], and recognizing the biological heterogeneity intrinsic to GAc beyond the Lauren classification is a logical step forward in improving patient survival.

## Supporting information

S1 Data Tabulated data corresponding to Fig 1. (XLSX)
